# Empathy and alexithymia in essential tremor

**DOI:** 10.3389/fpsyt.2026.1794068

**Published:** 2026-04-16

**Authors:** Maria Grazia Maggio, Antonina Luca, Calogero Edoardo Cicero, Concetta D’Agate, Lilla Bonanno, Amelia Rizzo, Gianluca Latino, Angelo Quartarone, Rocco Salvatore Calabrò, Alessandra Nicoletti

**Affiliations:** 1 Nerorehabilitation Unit, Istituto di Ricovero e Cura a Carattere Scientifico (IRCCS), Centro Neurolesi “Bonino Pulejo”, Messina, Italy; 2 Department of Medicine and Surgery, Kore University of Enna, Cittadella Universitaria, Enna, Italy; 3 Department “G.F. Ingrassia”, Section of Neurosciences, University of Catania, Catania, Italy; 4 Department of Clinical and Experimental Medicine, University of Messina, Messina, Italy

**Keywords:** movement disorders, neuropsychology, neurorehabilitation, neuroscience, social cognition

## Abstract

**Background:**

Social cognition is increasingly recognized as part of the non-motor phenotype of essential tremor (ET). Available ET evidence suggests selective alterations in some socio-cognitive domains, whereas findings on self-reported empathy and alexithymia remain limited and inconsistent.

**Objectives:**

This cross-sectional study aimed to evaluate empathy and alexithymia in patients with ET compared with healthy controls (HC), and to explore their associations with global cognition and with each other.

**Methods:**

Forty ET patients and 40 HC underwent the Italian versions of the Montreal Cognitive Assessment (MoCA), the short Empathy Quotient (EQ-short), and the Toronto Alexithymia Scale (TAS-20).

**Results:**

ET patients had significantly lower MoCA scores than HC (22.1 ± 4.1 vs. 25.3 ± 3.2, p<0.001), whereas no between-group differences emerged for EQ-short or TAS-20 scores. In ET, MoCA was not significantly associated with empathy or alexithymia measures. In HC, higher MoCA scores were associated with greater emotional reactivity. Exploratory bivariate analyses suggested inverse associations between social skills and alexithymia in ET, but only the adjusted ET models remained significant.

**Conclusion:**

Our findings do not support a group-level deficit in self-reported empathy or alexithymia in ET. Rather, they suggest that socio-emotional functioning may be largely preserved at the group level, while the relationship between social skills and emotional self-description may differ in ET.

## 1 Introduction

Emotional functioning involves the ability to perceive, interpret, and regulate affective states in oneself and others, allowing adaptive emotional and social behavior (1). Within this framework, social cognition (SC) comprises the neurocognitive processes that regulate adaptive behavioral responses to social and emotional cues (2–4). Among its core domains, empathy encompasses understanding others’ mental states (cognitive empathy) and sharing their emotional experiences (affective empathy) (5–7). Beyond SC, individual differences in emotional awareness also influence interpersonal functioning. Alexithymia, defined as difficulty in identifying and describing one’s emotions, represents a multidimensional construct associated with impaired emotional regulation and reduced empathy (8–11). In neurological conditions, acquired alexithymia may emerge following brain damage or neurodegenerative processes that alter limbic-cortical and fronto-striatal networks involved in emotional processing (12). Previous research has mostly focused on Parkinson’s disease, showing alterations in empathy/theory of mind and alexithymic traits as part of the non-motor phenotype (13, 14).

However, less is known about whether similar socioemotional alterations also occur in essential tremor (ET). Available ET evidence suggests a selective and heterogeneous pattern rather than a generalized socio-affective impairment. In case-control studies, ET patients showed poorer performance on cognitive theory of mind tasks, whereas affective theory of mind appeared relatively preserved compared with controls (15, 16). By contrast, studies specifically using the TAS-20 reported higher alexithymia levels in ET, including findings that remained after controlling for or excluding clinically relevant depression and anxiety; alexithymia in ET has also been linked to cognitive and brain microstructural correlates (17–19). More broadly, alexithymia is theoretically relevant to social cognition because systematic review evidence indicates that alexithymia is often associated with poorer theory of mind, especially when tasks require emotion recognition (11).

Recent neuroimaging studies suggest that ET extends beyond cerebello-thalamo-cortical motor circuits and involves cerebellar-limbic and cerebello-cortical networks related to emotional and cognitive functions (20–22). These findings are consistent with the growing view of the cerebellum as a hub for integrating sensorimotor, affective, and social information (23, 24). Taken together, the available evidence suggests that ET may involve socio-affective heterogeneity rather than a uniform deficit and provides a rationale for examining self-perceived empathy and alexithymia as complementary aspects of socio-emotional functioning.

Despite the clinical relevance of socio-emotional skills for interpersonal functioning and quality of life, these components are often overlooked in routine assessment and rehabilitation of individuals with ET. We selected empathy and alexithymia because they capture complementary aspects of self-perceived socio-affective functioning and emotional self-awareness, thereby providing information that complements rather than duplicates the existing ET literature based mainly on performance-based theory-of-mind paradigms. However, an important unresolved issue is whether self-perceived socio-affective functioning reflects the alterations observed in performance-based social cognition tasks in ET. Self-report measures and performance-based assessments may capture partially distinct constructs, reflecting subjective awareness and objective cognitive processing, respectively. This distinction raises the question of whether socio-affective alterations in ET are consistently detectable across different levels of assessment or may remain partially unrecognized at the subjective level. Accordingly, the aims of this observational study were to evaluate empathy and alexithymia in ET and in a group of healthy controls (HC), and to explore possible correlations among global cognition, empathy, and alexithymia in both groups.

## 2 Methods

### 2.1 Study population

Subjects who attended the “Parkinson’s disease and movement disorders Centre” of the University of Catania, Italy, between March 2022 and July 2023 were enrolled in the study.

Inclusion criteria were: i) diagnosis of essential tremor according to the Consensus Statement on Tremor of the Movement Disorder Society (25); ii) age between 40 and 80 years; iii) at least 5 years of education; and iv) the ability to provide written informed consent. Exclusion criteria were the presence of psychiatric disorders (major depression, psychosis, or anxiety disorders) and dementia. Exclusion criteria were operationalized on the basis of documented clinical diagnoses in the medical record and clinician judgment at enrollment; formal rating scales for depression, anxiety, or dementia were not systematically administered within the study protocol. In addition, healthy adult volunteers were recruited to obtain a control group broadly comparable in age and sex from patients’ partners and friends and hospital staff. HC were excluded if they reported a history of neurological disease, major psychiatric illness, or clinically evident cognitive or functional decline at enrollment.

### 2.2 Ethics

The study was conducted in accordance with the ethical standards of the 1964 Helsinki Declaration and its later amendments. Data from patients were collected during routine clinical assessments, while HC underwent non-invasive cognitive and behavioral evaluations. All data were pseudo-anonymized prior to analysis, and all participants provided written informed consent for the use of their data for research and publication purposes.

### 2.3 Procedures

Participation in the study was voluntary. All enrolled participants were evaluated individually by a psychologist in a quiet room using the same standardized neuropsychological battery. All measures were administered in their Italian versions. The Montreal Cognitive Assessment (MoCA) was used as a brief measure of global cognitive efficiency (26); Italian normative studies indicate that age and education significantly influence MoCA performance and support demographically informed interpretation (27, 28). Empathy was assessed with the EQ-short, originally developed by Wakabayashi et al. (29) and supported in Italian samples by studies showing good validity, a stable three-factor structure, and adequate reliability of both the EQ and its short Italian version (30, 31). Alexithymia was assessed with the TAS-20 (10), whose Italian version has demonstrated adequate factorial validity and reliability (32). We focused on empathy and alexithymia because the aim of the study was to investigate self-perceived socio-affective functioning and emotional self-awareness; thus, these measures complement rather than replicate the existing ET literature based mainly on performance-based theory-of-mind tasks. For interpretive purposes, standard TAS-20 categories were defined as no alexithymia (≤50), borderline alexithymia (51–60), and alexithymia (≥61) (10).

### 2.4 Statistical analysis

Data were entered into a dedicated database and analyzed using Stata Statistical Software, Release 18 (StataCorp LLC, College Station, TX, USA). Categorical variables were summarized as number and percentage and compared using the chi-square test or Fisher’s exact test, as appropriate. Continuous variables were summarized as mean and standard deviation or median and interquartile range, depending on distribution. Normality was assessed using the Shapiro-Wilk test. Between-group comparisons were performed using the independent-samples t-test for normally distributed variables and the Mann-Whitney U test for non-normally distributed variables. To aid interpretation of non-significant between-group findings, standardized effect sizes (Hedges g) with 95% confidence intervals were also calculated. Spearman’s rank correlation coefficients were used to explore associations between MoCA and socio-affective measures, and between empathy and alexithymia domains, separately in ET and HC. To limit type I error due to multiple correlation testing, Benjamini-Hochberg false discovery rate (FDR) correction was applied within each family of correlations. Multivariable linear regression models were then used as secondary adjusted analyses for clinically relevant associations, controlling *a priori* for age, education, and MoCA score. Because MoCA performance in Italian samples is influenced by age and education, MoCA was treated as a continuous covariate rather than using a single raw cut-off as an exclusion criterion. No specific MoCA cut-off was applied for participant selection, as the study aimed to preserve variability in cognitive performance within both groups and to explore its potential role as a clinically relevant dimension (27, 28). Two-sided p-values <0.05 were considered statistically significant. A sensitivity analysis was performed excluding HC participants with MoCA scores <23, in order to assess the potential impact of non-normative cognitive performance on the results.

## 3 Results

Forty ET [16 (40%) women, mean age 71.0 ± 4.9 years, mean education 9.4 ± 4.2 years, mean disease duration 5.6 ± 1.6] and 40 HC [22 (55%) women, mean age 70.5 ± 8.1 years, mean education 11.3 ± 4.7 years] were enrolled. Comparing the two groups, no statistically significant differences in sex (p=0.179), age (p=0.305), or education (p=0.084) were found. Patients with ET had a significantly lower MoCA score than HC (22.1 ± 4.1 vs. 25.3 ± 3.2, p<0.001). The distribution of MoCA scores showed partial overlap between groups (ET range: 11–28; HC range: 15–30), without evidence of extreme outliers, indicating some heterogeneity in cognitive performance across both groups. To address the potential influence of lower cognitive performance in HC, a sensitivity analysis excluding HC participants with MoCA scores <23 (n=7) was performed. The overall pattern of results remained unchanged, confirming the absence of significant between-group differences in empathy and alexithymia measures.

No statistically significant between-group differences emerged for EQ-CE, EQ-ER, EQ-SS, or TAS-20 scores (**Table 1**). Standardized effect sizes were small for empathy and alexithymia measures (absolute Hedges g range 0.03-0.25), whereas the between-group difference in MoCA was moderate-to-large (Hedges g=-0.86; 95% CI -1.32 to -0.41).

**Table 1:** Neuropsychological characteristics of the sample.

Test/scales	ET (n=40)	HC (n=40)	p-value
MoCA	22.1 ± 4.1	25.3 ± 3.2	**<0.001^¥^**
EQ-CE	6.5 ± 2.6	6.8 ± 2.2	0.674^¥^
EQ-ER	6.8 ± 2.9	7.1 ± 2.1	0.546^+^
EQ-SS	5.7 ± 2.5	5.2 ± 2.4	0.392^+^
TAS-20 DIF	14.2 ± 4.8	13.1 ± 3.9	0.247^+^
TAS-20 DDF	16.5 ± 5.9	17.1 ± 5.3	0.666^+^
TAS-20 EOT	22.9 ± 4.9	23.8 ± 5.8	0.446^+^
TAS-20 TOT	53.7 ± 11.5	54.0 ± 11.8	0.901^+^

Data are expressed as number and percentage or mean and standard deviation. ¥ Mann-Whitney U test; + independent-samples t-test. ET, Essential Tremor; HC, Healthy Controls; MoCA, Montreal Cognitive Assessment; EQ-CE, Empathy Quotient-Cognitive Empathy; EQ-ER, Empathy Quotient-Emotional Reactivity; EQ-SS, Empathy Quotient-Social Skills; TAS-20 DIF, Toronto Alexithymia Scale-Difficulty Identifying Feelings; TAS-20 DDF, Toronto Alexithymia Scale-Difficulty Describing Feelings; TAS-20 EOT, Toronto Alexithymia Scale-Externally Oriented Thinking; TAS-20 TOT, Toronto Alexithymia Scale-Total Score. Bold values indicate statistically significant results (p < 0.05).

Concerning the relationship between cognitive performance (MoCA) and socio-affective measures, in the ET group no statistically significant correlations were observed between MoCA and EQ-CE (rho=0.190; p=0.239), EQ-ER (rho=0.136; p=0.400), EQ-SS (rho=0.154; p=0.341), TAS-20 DIF (rho=0.109; p=0.499), TAS-20 DDF (rho=0.037; p=0.816), TAS-20 EOT (rho=0.027; p=0.864), or TAS-20 total score (rho=0.015; p=0.926). After FDR correction across these seven ET correlations, none remained significant.

By contrast, in HC a positive correlation was found between MoCA and EQ-ER (rho=0.403; p=0.001), whereas correlations with EQ-CE (rho=0.257; p=0.108) and EQ-SS (rho=0.307; p=0.051) were not significant. No statistically significant correlations were observed between MoCA and alexithymia measures in HC. After FDR correction across the three reported MoCA-EQ correlations in HC, the MoCA-EQ-ER association remained significant (q=0.003).

**Figure 1:**
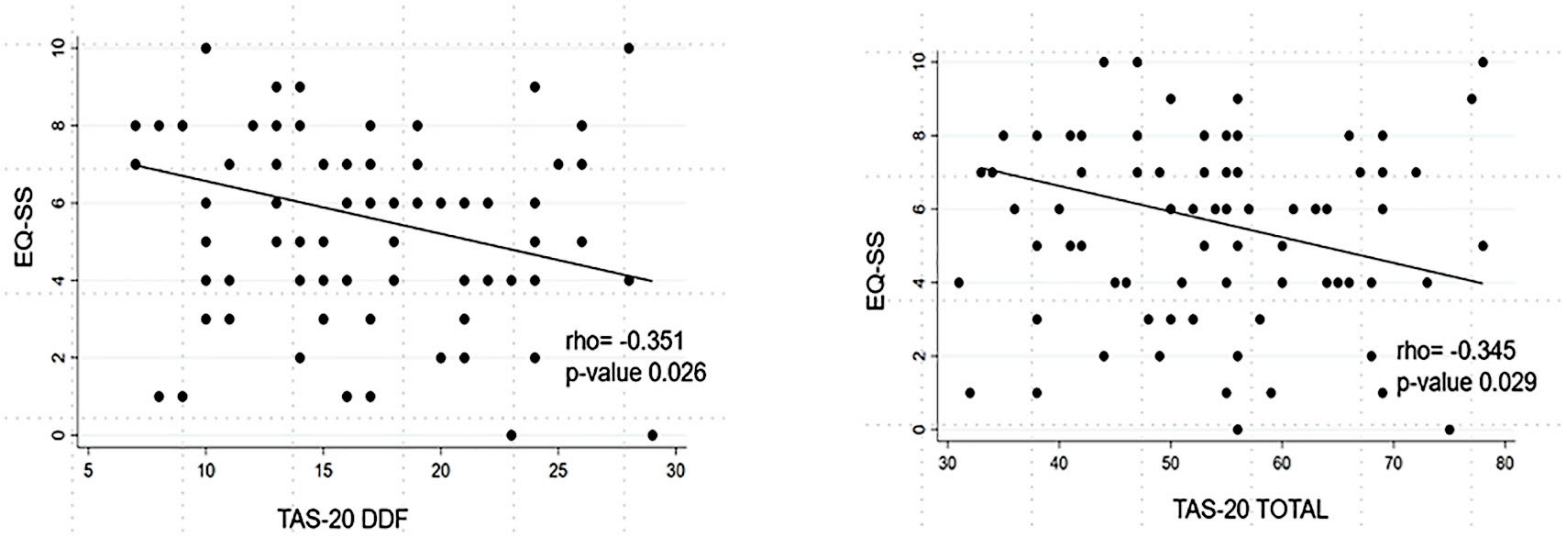
Exploratory unadjusted correlations between EQ-SS and TAS-20 DDF and total score in ET. ET, Essential Tremor; HC, Healthy Controls; EQ-CE, Empathy Quotient-Cognitive Empathy; EQ-ER, Empathy Quotient-Emotional Reactivity; EQ-SS, Empathy Quotient-Social Skills; TAS-20 DIF, Toronto Alexithymia Scale-Difficulty Identifying Feelings; TAS-20 DDF, Toronto Alexithymia Scale-Difficulty Describing Feelings; TAS-20 EOT, Toronto Alexithymia Scale-Externally Oriented Thinking; TAS-20 TOT, Toronto Alexithymia Scale-Total Score.

Concerning the relationship between empathy and alexithymia, exploratory bivariate analyses suggested inverse associations between EQ-SS and TAS-20 DDF (rho=-0.351; p=0.026) and TAS-20 total score (rho=-0.345; p=0.029) in the ET group, and a positive association between EQ-SS and TAS-20 EOT (rho=0.386; p=0.013) in the HC group (**Table 2**; **Figure 1**). However, after FDR correction across the 12 empathy-alexithymia correlations within each group, these associations were no longer statistically significant (ET q=0.174 for both EQ-SS/TAS-20 DDF and EQ-SS/TAS-20 total; HC q=0.156 for EQ-SS/TAS-20 EOT).

**Table 2:** Spearman’s correlations between empathy and alexithymia in the two groups.

Test/scales	TAS-20 DIF		TAS-20 DDF		TAS-20 EOT		TAS-20 total score	
	rho	p-value	rho	p-value	rho	p-value	rho	p-value
ET group								
EQ-CE	0.084	0.604	-0.185	0.250	-0.154	0.340	-0.169	0.293
EQ-ER	0.056	0.729	0.136	0.402	-0.018	0.909	0.080	0.620
EQ-SS	-0.188	0.243	**-0.351**	**0.026**	-0.128	0.429	**-0.345**	**0.029**
HC group								
EQ-CE	0.093	0.566	0.251	0.117	0.060	0.713	0.145	0.371
EQ-ER	-0.038	0.814	0.128	0.431	0.095	0.556	0.044	0.783
EQ-SS	0.124	0.445	0.099	0.543	**0.386**	**0.013**	0.242	0.131

ET, Essential Tremor; HC, Healthy Controls; EQ-CE, Empathy Quotient-Cognitive Empathy; EQ-ER, Empathy Quotient-Emotional Reactivity; EQ-SS, Empathy Quotient-Social Skills; TAS-20 DIF, Toronto Alexithymia Scale-Difficulty Identifying Feelings; TAS-20 DDF, Toronto Alexithymia Scale-Difficulty Describing Feelings; TAS-20 EOT, Toronto Alexithymia Scale-Externally Oriented Thinking; TAS-20 TOT, Toronto Alexithymia Scale-Total Score. Benjamini-Hochberg FDR-adjusted q-values for the nominally significant correlations were 0.174 for ET EQ-SS/TAS-20 DDF, 0.174 for ET EQ-SS/TAS-20 total, and 0.156 for HC EQ-SS/TAS-20 EOT. Bold values indicate statistically significant results (p < 0.05).

In secondary multivariable linear regression models adjusting for age, education, and MoCA score, lower EQ-SS remained independently associated with higher TAS-20 DDF (coeff.=-0.15; 95% CI -0.28 to -0.01; p=0.030) and higher TAS-20 total score (coeff.=-0.07; 95% CI -0.14 to -0.01; p=0.032) in the ET group. These two associations also survived FDR correction across the three regression models (q=0.048 for both). By contrast, the HC association between EQ-SS and TAS-20 EOT was not confirmed (coeff. = 0.11; 95% CI -0.02 to 0.24; p=0.116).

## 4 Discussion

This study explored self-reported socio-affective functioning in ET compared with HC. Although ET has traditionally been considered a pure motor disorder, accumulating evidence indicates the presence of non-motor features including cognitive and affective changes (33, 34). In our sample, ET patients showed lower global cognitive performance on the MoCA but did not differ from HC in self-reported empathy or alexithymia.

At the group level, therefore, our data do not support a generalized socio-affective deficit in ET as captured by the EQ-short and TAS-20. Rather, they suggest that socio-emotional functioning may be largely preserved at the group level despite lower global cognition. This pattern is partly consistent with ET studies reporting impaired cognitive theory of mind but relatively preserved affective theory of mind (15, 16), and only partly consistent with prior TAS-20 studies reporting higher alexithymia levels in ET (17–19). Differences between self-report and performance-based measures, sample characteristics, the relatively modest raw MoCA mean in HC, and the absence of direct mood measures may help explain these discrepancies. A more detailed comparison with previous ET studies helps clarify these partially divergent findings. Prior investigations have consistently reported impairments in cognitive theory of mind in ET, with relatively preserved affective components, based on performance-based tasks such as the RMET or false-belief paradigms (15, 16). In contrast, our study did not observe group-level differences in self-reported empathy or alexithymia. This discrepancy may reflect fundamental differences between assessment approaches. Performance-based tasks capture the ability to infer mental states under structured conditions, relying on executive and inferential processes, whereas self-report measures primarily reflect subjective awareness and appraisal of one’s own socio-affective functioning. As a result, deficits detectable at the performance level may not necessarily be perceived or reported by patients, particularly in conditions characterized by subtle or heterogeneous socio-cognitive alterations. Similarly, previous studies using the TAS-20 have reported higher levels of alexithymia in ET (17–19), including findings that persist after controlling for depression and anxiety. The absence of between-group differences in our sample may be related to differences in sample characteristics and study design. In particular, our HC group showed some variability in cognitive performance, which may have attenuated subtle group differences, although sensitivity analyses confirmed that this did not substantially influence the results. In addition, mood variables were not directly assessed in the present study, whereas prior literature suggests that depression and anxiety may contribute significantly to alexithymia scores in ET. Taken together, these findings do not necessarily contradict previous evidence but rather suggest that socio-affective functioning in ET may manifest differently depending on the level of assessment. The ET-specific association between lower social skills and poorer emotional self-description should be interpreted as a secondary within-group signal rather than as evidence of a group-level deficit. Importantly, the corresponding unadjusted bivariate correlations were attenuated after correction for multiple testing, although the inverse association remained detectable in adjusted models. Accordingly, these findings are best considered hypothesis-generating and in need of replication.

The absence of significant associations between MoCA and socio-affective measures in ET, together with the positive MoCA-EQ-ER association in HC, suggests that the relationship between global cognition and self-reported socio-affective functioning is not straightforward in ET. This interpretation is compatible with the growing view that ET involves distributed cerebello-thalamo-cortical and cerebello-limbic networks extending beyond motor circuits (20–24).

Aging and education remain relevant to interpretation because socio-cognitive and socio-affective processes may be differentially affected in older adults. Recent reviews indicate that aging is associated with heterogeneous changes in social cognition, with theory of mind and some cognitive-empathy components appearing more vulnerable than affective empathy, whereas higher educational level may mitigate age-related differences in cognitive empathy (35, 36). In addition, depression and anxiety are clinically relevant non-motor manifestations of ET and may influence socio-affective outcomes (34, 37). Although age, education, and MoCA were included in adjusted analyses, residual confounding cannot be excluded because mood measures were not directly administered.

Future studies should combine self-report, performance-based social cognition tasks, mood assessment, and multimodal biomarkers to clarify whether ET is characterized mainly by preserved socio-affective self-perception, altered socio-cognitive performance, or specific subgroups with greater emotional vulnerability.

### 4.1 Clinical implications

Although empathy and alexithymia did not differ at the group level between ET and HC, selected patients may still experience subtle socio-emotional difficulties affecting communication and interpersonal functioning. Accordingly, socioemotional screening may be most useful in ET patients who report relational or emotional communication difficulties rather than as evidence of a universal deficit. Multidisciplinary care pathways may benefit from integrating socioemotional assessment alongside motor and cognitive evaluation.

### 4.2 Strengths and limitations

This study addresses an underexplored non-motor dimension of ET and includes a control group broadly comparable in age and sex, standardized self-report measures, and adjusted analyses. Several limitations warrant emphasis. First, the sample was relatively small, and no *a priori* sample-size calculation was performed; therefore, the study was not powered to demonstrate equivalence or exclude small between-group effects. Second, the cross-sectional design precludes causal inference. Third, exclusion of psychiatric disorders was based on clinical history and clinician judgment rather than structured mood scales, and depression/anxiety were not directly measured despite their known relevance in ET. Fourth, HC displayed a relatively modest raw MoCA mean; although MoCA interpretation in Italy should rely on age- and education-adjusted norms rather than a universal raw cut-off, this characteristic may have attenuated subtle between-group differences. Although HC were screened based on clinical criteria, some participants showed relatively low MoCA scores. However, sensitivity analyses excluding HC with MoCA <23 confirmed that the overall pattern of findings remained unchanged, suggesting that this factor did not substantially influence the results.

Fifth, the study relied on self-report instruments and did not include performance-based facial emotion recognition or theory-of-mind tasks, limiting direct comparability with prior ET literature. Finally, the single-center design and recruitment of HC from partners, friends, and hospital staff may limit generalizability.

## 5 Conclusions

This study did not show between-group differences in self-reported empathy or alexithymia between ET patients and HC, but it suggests that the relationship between social skills and emotional self-description may differ in ET. These findings support a nuanced view of ET as a disorder with possible socio-affective heterogeneity beyond motor symptoms while underscoring the need for larger, multimodal studies including mood measures, demographically informed cognitive screening, and performance-based social cognition tasks.

## Data Availability

The raw data supporting the conclusions of this article will be made available by the authors, without undue reservation.
